# Forensic efficiency estimate and phylogenetic analysis for Chinese Kyrgyz ethnic group revealed by a panel of 21 short tandem repeats

**DOI:** 10.1098/rsos.172089

**Published:** 2018-06-13

**Authors:** Yuxin Guo, Chong Chen, Tong Xie, Wei Cui, Haotian Meng, Xiaoye Jin, Bofeng Zhu

**Affiliations:** 1 Key Laboratory of Shaanxi Province for Craniofacial Precision Medicine Research, College of Stomatology, Xi'an Jiaotong University, Xi'an 710004, People's Republic of China; 2 Clinical Research Center of Shaanxi Province for Dental and Maxillofacial Diseases, College of Stomatology, Xi'an Jiaotong University, Xi'an 710004, People's Republic of China; 3 College of Medicine and Forensics, Xi'an Jiaotong University Health Science Center, Xi'an 710061, People's Republic of China; 4 Department of Forensic Genetics, School of Forensic Medicine, Southern Medical University, Guangzhou 510515, People's Republic of China

**Keywords:** non-CODIS STRs, Chinese Kyrgyz, forensic, population genetics

## Abstract

Short tandem repeats (STRs) with a high level of polymorphisms and convenient detection method play an indispensable role in human population and forensic genetics. Recently, we detected the 21 autosomal non-combined DNA index system (non-CODIS) STR loci in a Kyrgyz ethnic group, calculated their forensic parameters and analysed its genetic relationships with reference populations from China. In total, 168 alleles were observed at 21 non-CODIS STRs with corresponding allelic frequencies from 0.0016 to 0.4788. No significant deviations at these STRs were observed from the Hardy–Weinberg equilibrium. The values of cumulative power of discrimination and probability of exclusion for all the 21 non-CODIS STRs were 0.99999999999999999998835 and 0.9999994002, respectively. Furthermore, the analyses of phylogenetic trees, genetic distances and interpopulation differentiations demonstrated that the Kyrgyz group had relatively close genetic relationships with the Uygur and Kazak groups. These 21 non-CODIS STRs were characterized by high genetic diversities in the Kyrgyz group and could be applied as a robust tool for individual identification and kinship testing in forensic sciences.

## 1. Introduction

Short tandem repeats (STRs), as the most common genetic markers, well widespread in the human genome, have had a broad range of applications in DNA profiling of routine casework (especially in individual identification and paternity testing) for several decades [1–4]. Although many commercial kits of autosomal STRs have been developed [5–7], most of them contain the 13 overlapping loci researched by the combined DNA index system (CODIS) [8]. In forensic practice, to solve some disputed kinship testing, such as the duo parentage analysis which lacked the sample from father or mother, usually needs more non-CODIS STR loci to achieve the identifying criterion. In addition, the mutation rates of STR loci are relatively high; for this reason, the result of parentage testing tends to be complex if even one or two mismatches occur between parent and offspring. As a result, more non-CODIS STR loci are needed as a supplementary. However, the kits mentioned above are not suitable for using together as complements to maximize the distinguishability [9]. Therefore, it is meaningful to select more STR loci without the overlapping 13 CODIS core loci in the forensic applications, especially in the complicated kinship cases and missing person investigations.

The Kyrgyz group is one of the 56 ethnic groups in China and comprises a population of 186 708, which is mainly spread over Kizilsu Kirghiz Autonomous Prefecture of Xinjiang Uygur Autonomous Region, with small proportions distributed in different regions of Xinjiang; only a few remain dwelling in Fuyu County, Heilongjiang Province (all the data were taken from the Sixth National Population Census of the People's Republic of China) (http://www.stats.gov.cn/tjsj/pcsj/rkpc/6rp/indexch.htm). Their language belongs to the Altai language family, and the written language which they use today was created based on the Arabic alphabet.

There have been quite a few research works on the Uygur, Kazak and other ethnic groups in Xinjiang [10–14], but very few available regarding the Kyrgyz from China. To enrich the population genetic data library and explore the genetic background of the Kyrgyz, a panel of 21 non-CODIS STR loci was employed to analyse the individuals of the Chinese Kyrgyz group by comparing them with 11 previously published populations.

## 2. Material and methods

### 2.1. Sample collection and DNA extraction

Peripheral blood was extracted from 307 unrelated healthy individuals dwelling in the Kizilsu Kirghiz Autonomous Prefecture of Xinjiang Uygur Autonomous Region for more than three generations. Written informed consent was obtained from every participant. After collection of the peripheral blood, a small part of the blood, which was spread on a fresh filter and allowed to dry at room temperature, was made into a bloodstain for long-term conservation, and the remaining part was frozen for storage. This study was carried out according to the humane and ethical research principles approved by the ethical committee of Xi'an Jiaotong University Health Science Center, China (no. XJTULAC201). DNA was extracted from the bloodstain mentioned above by the Chelex-100 method [15].

### 2.2. Polymerase chain reaction amplification and short tandem repeat typing

The 21 non-CODIS STR loci were amplified simultaneously in a single polymerase chain reaction (PCR) system by the AGCU 21+1 fluorescence amplification reagents (AGCU ScienTech Incorporation, Wuxi, Jiangsu, China) with a GeneAmp PCR System 9700 Thermal Cycler (Applied Biosystems, Foster City, CA, USA). The PCR conditions could be obtained from the developmental validation of the reagent [9]. STR genotyping was performed on an ABI PRISM 3130 Genetic Analyzer (Applied Biosystems) and analysed by the Genemapper ID 3.2 software (Applied Biosystems).

### 2.3. Quality control

The study was in accordance with ISFG recommendations by Schneider [16] on the analysis of DNA polymorphisms.

### 2.4. Statistical analyses

A modified Powerstat v. 1.2 spreadsheet [17] was used to compute the allelic frequencies and forensic parameters including matching probability (MP), power of discrimination (PD), probability of exclusion (PE), observed heterozygosity (HO), polymorphism information content (PIC), typical paternity index (TPI) and exact tests of the Hardy–Weinberg Equilibrium (HWE). Meanwhile, the values of expected heterozygosity (HE), cumulative power of discrimination (CPD) and cumulative probability of exclusion (CPE) were calculated with formulas directly. Linkage disequilibrium (LD) exact tests of each pair of STR loci and pairwise *F*_st_ values were estimated by Genepop v. 4.0.10 (http://genepop.curtin.edu.au/). Population structure analyses were conducted among the studied Kyrgyz ethnic group and other populations previously published using the Structure program v. 2.2 (http://pritch.bsd.uchicago.edu/structure.html), and the plot of optimum *K* (*K *= 6) determined by Structure Harvester v. 0.6.94 [18] was portrayed using Distruct v. 1.1 (https://web.stanford.edu/group/rosenberglab/distruct.html). The neighbour-joining (NJ) tree was constructed based on the *D*_A_ values computed with the Dispan program by MEGA v. 6.06 (http://megasoftware.net/) and another tree was constructed by Phylip v. 3.69 based on allelic frequencies. PASW Statistics v. 18 (http://www.winwrap.com) was used to create a multidimensional scaling (MDS) plot based on pairwise *F*_st_ values. In addition, locus-by-locus *F*_st_ and *p-*values between the Kyrgyz group and the other compared populations were calculated by Arlequin software v. 3.1 (http://cmpg.unibe.ch/software/arlequin3).

## 3. Results and discussion

### 3.1. Genetic polymorphism analyses of 21 non-combined DNA index system loci

Allelic frequencies and forensic parameters of the 21 non-CODIS STR loci are listed in tables 1 and 2, respectively. There were 168 alleles observed with corresponding allelic frequencies from 0.0016 to 0.4788 in the group. No significant deviations (*p *> 0.05) were observed from the HWE in these 21 STR loci. The maximum values of PD, PE, PIC and TPI were 0.9494, 0.7081, 0.8090 and 3.4886, respectively, which were all observed at locus D19S433. On the contrary, the minimum values all observed at locus D1S1627 were 0.7617, 0.2565, 0.51044 and 1.1629, respectively. In addition, the MP values ranged from 0.0506 at locus D19S433 to 0.2383 at locus D1S1627. HO values were in a range from 0.8567 (D19S433) to 0.5700 (D1S1627), while the HE values ranged from 0.8284 (D19S433) to 0.5955 (D1S1627). The CPD and CPE values of all these 21 STR loci were 0.99999999999999999998844 and 0.9999993992, respectively. The results mentioned above indicated that the panel of non-CODIS 21 STR loci could be used as a sensitive and accurate tool in routine forensic caseworks of the studied Kyrgyz group.

**Table 1.** The allelic frequencies for the 21 STR loci in the Kyrgyz group (*n* = 307).

alleles	D10S1248	D10S1435	D11S4463	D12ATA63	D14S1434	D17S1301	D18S853	D19S433	D1GATA113	D1S1677	D20S482	D22S1045	D2S1776	D2S441	D3S4529	D4S2408	D5S2500	D6S1017	D6S474	D9S1122	D1S1627
7.0		0.0033				0.0163			0.4267				0.0033								
8.0	0.0016	0.0033				0.0016	0.0016		0.0147				0.0195			0.2590		0.2362			
9.0		0.0033	0.0016			0.0163							0.0798			0.3094		0.0114			
9.1														0.0033							
10.0		0.0179			0.1629	0.0521	0.098		0.0033		0.0440	0.0081	0.1107	0.2264		0.2573		0.3844		0.0684	0.0505
11.0	0.0033	0.1156	0.0326		0.1661	0.2980	0.3925	0.0033	0.1629	0.0016	0.0293	0.2606	0.2801	0.4577		0.1466		0.0375		0.1743	0.0293
11.2								0.0016													
11.3														0.0212							
12.0	0.0537	0.3844	0.0505	0.3371	0.0179	0.3762	0.0700	0.0521	0.3534	0.0293	0.0700	0.0098	0.3681	0.0993	0.0163	0.0277		0.2590		0.2801	0.0130
12.2		0.0016						0.0065													
12.3														0.0016							
13.0	0.2459	0.2655	0.2866	0.0798	0.3534	0.1873	0.2150	0.2296	0.0391	0.1678	0.2117	0.0130	0.1124	0.0195	0.2345		0.0016	0.0700	0.0049	0.3844	0.4788
13.2		0.0065						0.0521													
14.0	0.2590	0.1889	0.2834	0.0244	0.2752	0.0505	0.2476	0.2785		0.4463	0.4072	0.0358	0.0228	0.1384	0.2068		0.2704	0.0016	0.3518	0.0651	0.4137
14.2								0.0749													
15.0	0.2866	0.0065	0.2134	0.0554	0.0195	0.0016	0.0586	0.0977		0.2720	0.1629	0.2606	0.0033	0.0326	0.3485				0.3306	0.0130	0.0130
15.2								0.1336													
16.0	0.1010	0.0033	0.1075	0.0847	0.0049		0.0049	0.0261		0.0749	0.0700	0.2427			0.1661				0.1254	0.0147	0.0016
16.2								0.0391													
17.0	0.0489		0.0244	0.3681						0.0081	0.0049	0.1401			0.0277		0.3583		0.1368		
17.2								0.0049													
18.0				0.0489								0.0261					0.2524		0.0505		
19.0												0.0033					0.0098				
20.0				0.0016													0.0472				
23.0																	0.0423				
24.0																	0.0179

**Table 2.** The statistical parameters for the 21 STR loci in the Kyrgyz group (*n* = 307). (MP, matching probability; PD, power of discrimination; PIC, polymorphism information content; PE, probability of exclusion; TPI, typical paternity index; HO, observed heterozygosity; HE, expected heterozygosity; *p*, probability values of exact tests for Hardy–Weinberg equilibrium.)

loci	MP	PD	PIC	PE	TPI	HO	HE	*p*-values
D10S1248	0.0870	0.9130	0.7391	0.5656	2.2910	0.7818	0.7745	0.8111
D10S1435	0.1204	0.8796	0.6888	0.4973	1.9430	0.7427	0.7327	0.7153
D11S4463	0.0899	0.9101	0.7416	0.6259	2.6930	0.8143	0.7766	0.1208
D12ATA63	0.1086	0.8914	0.6904	0.4810	1.8720	0.7329	0.7314	0.9861
D14S1434	0.1109	0.8891	0.7020	0.5140	2.0197	0.7524	0.7449	0.7861
D17S1301	0.1184	0.8816	0.6843	0.4389	1.7056	0.7068	0.7291	0.3621
D18S853	0.1196	0.8804	0.6867	0.4492	1.7443	0.7134	0.7293	0.4816
D19S433	0.0506	0.9494	0.8090	0.7081	3.4886	0.8567	0.8284	0.2188
D1GATA113	0.1783	0.8217	0.6018	0.3896	1.5350	0.6743	0.6647	0.7536
D1S1677	0.1373	0.8627	0.6434	0.3709	1.4760	0.6612	0.6923	0.2227
D20S482	0.0950	0.9050	0.7181	0.4702	1.8274	0.7264	0.7502	0.3093
D22S1045	0.0842	0.9158	0.7495	0.6198	2.6466	0.8111	0.7828	0.2601
D2S1776	0.0966	0.9034	0.7181	0.4918	1.9188	0.7394	0.7540	0.5230
D2S441	0.1277	0.8723	0.6703	0.4338	1.6868	0.7036	0.7084	0.8188
D3S4529	0.0998	0.9002	0.7110	0.4441	1.7247	0.7101	0.7515	0.0788
D4S2408	0.1113	0.8887	0.7041	0.5424	2.1620	0.7687	0.7490	0.4470
D5S2500	0.1232	0.8768	0.6835	0.5028	1.9679	0.7459	0.7310	0.5695
D6S1017	0.1237	0.8763	0.6757	0.4338	1.6868	0.7036	0.7234	0.4195
D6S474	0.1290	0.8710	0.6848	0.5309	2.1027	0.7622	0.7296	0.2190
D9S1122	0.1131	0.8869	0.6924	0.4188	1.6330	0.6938	0.7346	0.0993
D1S1627	0.2383	0.7617	0.5144	0.2565	1.1629	0.5700	0.5955	0.3388

### 3.2. Linkage disequilibrium analyses of pairwise short tandem repeat loci

A previous study has verified that the physical distances among the non-CODIS 21 STRs were all greater than 10 Mb, that is, these loci were not linked with each other [9]. Also in this research, LD analyses should be conducted between each pair of STR loci before the next step in the analyses of population genetics and forensic science. Therefore, the LD tests in the 210 pairs of all the STR loci were performed by Genepop v. 4.0.10. After Bonferroni correction (*p *= 0.05/210 = 0.00024), no LD was observed in all the pairwise comparisons. Thus, the 21 STR loci could be considered as independent loci in the following analyses.

### 3.3. Structure analyses based on raw population data

**Figure 1.**
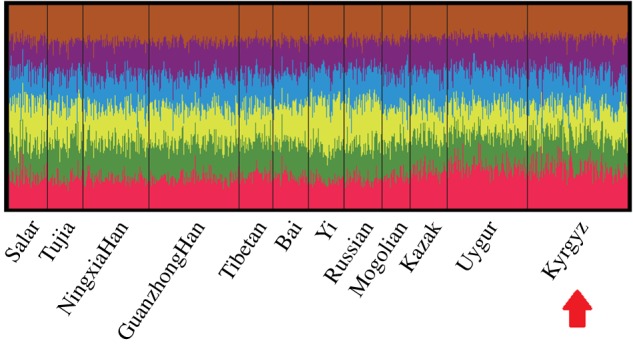
Population structure analyses was conducted by the raw data of Kyrgyz and the 11 reference groups (*K *= 6).

The population structure analyses were conducted on the basis of the raw population data of the same 21 STR loci between the studied Kyrgyz and other previously published populations by the Structre program. All the reference populations included nine populations from East Asia (Ningxia Han [19], Guanzhong Han [20], Tibetan [21], Bai [22], Yi [23], Russian [24], Salar [25], Tujia [26] and Mogolian [27]) and three populations from Central Asia (Chinese Kazak [28], Uygur [29] and the studied Kyrgyz). Each of *K *= 2–7 with 15 runs was carried out; then the optimum *K* was selected by Structure Harvester v. 0.6.94 and the results are shown in the electronic supplementary material, figure S1, suggesting that *K *= 6 was the most appropriate configuration. As shown in figure 1, all the analysed populations had a similar pattern of components distribution at *K *= 6, and proportions of the six colours were almost equal. Even the three populations (Kazak, Uygur and the studied Kyrgyz ethnic groups) from Central Asia were consistent with the nine East Asian populations roughly. Therefore, by the panel of 21 non-CODIS STR loci, no obvious population structure differentiation was observed among the Kyrgyz and the 11 reference populations mentioned above.

### 3.4. Interpopulation differentiations

The locus-by-locus *F*_st_ and *p-*values were calculated by the analysis of molecular variance (AMOVA) method between the Kyrgyz group and the 11 reference populations using Arlequin software v. 3.1. The results are given in table 3. Significant differences (*p *< 0.05) were detected between the Kyrgyz and the Yi group at 14 loci, followed by the Salar, Tibetan, Ningxia Han and Guanzhong Han groups at 11 loci, the Bai group at nine loci, the Tujia group at eight loci, the Russian and Mogolian groups at seven loci, the Uygur group at six loci, and finally the Kazak group at only one locus. Highest ethnic diversity was detected at the three loci (D10S1248, D12ATA63 and D1S1627) with significant differences observed between the Kyrgyz and the other 10 groups; in contrast, the lowest ethnic diversity was detected at locus D6S474 with no significant differences observed between the Kyrgyz group and the other reference groups.

**Table 3.** Locus-by-locus *F*_st_ and *p-*values of 21 STR loci between the Kyrgyz group and other reference groups. (*p-*values lower than 0.05 are in italics.)

loci	Salar		Tujia		Ningxia Han		Guanzhong Han		Tibetan		Bai		Yi		Russian		Mongolian		Kazak		Uygur	
	*F*_st_	*p-*values	*F*_st_	*p-*values	*F*_st_	*p-*values	*F*_st_	*p-*values	*F*_st_	*p-*values	*F*_st_	*p-*values	*F*_st_	*p-*values	*F*_st_	*p-*values	*F*_st_	*p-*values	*F*_st_	*p-*values	*F*_st_	*p-*values
D10S1248	0.0222	*0.0000*	0.0071	*0.0254*	0.0076	*0.0049*	0.0105	*0*.*0000*	0.0191	*0*.*0000*	0.0072	*0*.*0332*	0.0137	*0*.*0010*	0.0105	*0*.*0049*	0.0101	*0*.*0137*	−0.0004	0.8387	0.0054	*0*.*0127*
D10S1435	−0.0014	1.0000	0.0038	0.1633	0.0057	*0*.*0156*	0.0039	*0*.*0284*	−0.0008	0.8739	0.0009	0.5210	0.0275	*0*.*0000*	0.0056	0.0567	−0.0023	1.0000	0.0015	0.3930	0.0014	0.2796
D11S4463	0.0091	*0*.*0088*	0.0093	*0*.*0108*	0.0031	0.0929	0.0075	*0*.*0039*	0.0121	*0*.*0039*	0.0061	*0*.*0293*	0.0162	*0*.*0010*	−0.0018	1.0000	0.0075	*0*.*0381*	0.0089	*0*.*0166*	−0.0001	0.7654
D12ATA63	0.0085	*0*.*0147*	0.0172	*0*.*0000*	0.0069	*0*.*0068*	0.0096	*0*.*0020*	0.0164	*0*.*0059*	0.0110	*0*.*0098*	0.0204	*0*.*0010*	0.0135	*0*.*0039*	0.0137	*0*.*0059*	0.0005	0.6041	0.0001	0.6784
D14S1434	0.0146	*0*.*0020*	0.0465	*0*.*0000*	0.0186	*0*.*0000*	0.0079	*0*.*0010*	0.0264	*0*.*0000*	0.0128	*0*.*0078*	0.0434	*0*.*0000*	0.0182	*0*.*0010*	0.0269	*0*.*0000*	0.0056	0.0665	0.0118	*0*.*0000*
D17S1301	0.0237	*0*.*0000*	0.0026	0.2757	0.0205	*0*.*0000*	0.0135	*0*.*0000*	0.0083	*0*.*0205*	0.0197	*0*.*0000*	−0.0004	0.8094	0.0094	*0*.*0205*	0.0077	*0*.*0479*	−0.0022	1.0000	0.0030	0.0919
D18S853	−0.0012	0.9883	−0.0019	1.0000	−0.0009	0.9941	−0.0007	0.9932	0.0069	*0*.*0401*	0.0029	0.2317	−0.0013	0.9863	−0.0026	1.0000	−0.0003	0.7586	−0.0011	0.9560	0.0028	0.1124
D19S433	0.0008	0.5650	0.0028	0.1887	0.0043	*0*.*0186*	0.0017	0.1672	0.0023	0.2923	0.0024	0.2747	0.0009	0.5308	0.0037	0.1154	0.0059	*0*.*0489*	−0.0004	0.9257	0.0008	0.4379
D1GATA113	−0.0024	1.0000	0.0122	*0*.*0176*	0.0014	0.3197	0.0087	*0*.*0039*	0.0024	0.2698	0.0035	0.1828	0.0101	*0*.*0235*	−0.0018	1.0000	0.0000	0.6295	0.0005	0.5230	0.0044	*0*.*0479*
D1S1677	0.0083	*0*.*0313*	0.0047	0.1202	0.0103	*0*.*0010*	0.0102	*0*.*0039*	0.0015	0.4115	0.0083	*0*.*0362*	0.0063	0.0714	0.0018	0.3460	0.0006	0.5484	−0.0008	0.8788	0.0000	0.6833
D20S482	0.0023	0.2796	0.0048	0.1046	0.0033	0.0890	−0.0004	0.9003	0.0027	0.2483	0.0022	0.3011	0.0120	*0*.*0029*	0.0033	0.1554	−0.0002	0.8074	−0.0010	0.9736	0.0009	0.3900
D22S1045	0.0683	*0*.*0000*	0.0011	0.4428	−0.0009	0.9961	0.0000	0.7263	0.0105	*0*.*0029*	0.0002	0.7028	0.0842	*0*.*0000*	0.0884	*0*.*0000*	0.0040	0.1613	0.0008	0.4927	0.0044	*0*.*0156*
D2S1776	0.0063	*0*.*0411*	0.0011	0.4780	0.0004	0.6149	0.0010	0.3109	0.0036	0.1789	0.0029	0.2170	0.0074	*0*.*0244*	−0.0020	1.0000	0.0013	0.4497	0.0016	0.3744	−0.0009	1.0000
D2S441	0.0297	*0*.*0000*	0.0371	*0*.*0000*	0.0089	*0*.*0010*	0.0155	*0*.*0000*	0.0119	*0*.*0039*	0.0089	*0*.*0108*	0.0110	*0*.*0039*	0.0035	0.1593	0.0002	0.7087	0.0027	0.2278	0.0010	0.3646
D3S4529	0.0029	0.1984	−0.0027	1.0000	0.0007	0.4692	0.0031	0.0733	0.0051	0.0978	−0.0016	1.0000	−0.0021	1.0000	0.0062	0.0508	0.0075	0.0635	−0.0017	1.0000	−0.0009	1.0000
D4S2408	0.0066	*0*.*0362*	−0.0008	0.9081	0.0044	0.0547	−0.0001	0.7605	0.0092	*0*.*0225*	0.0008	0.5083	0.0238	*0*.*0000*	0.0060	0.0596	0.0059	0.0938	0.0028	0.2160	−0.0007	0.9668
D5S2500	0.0018	0.3197	0.0158	*0*.*0010*	0.0159	*0*.*0000*	0.0156	*0*.*0000*	0.0032	0.2248	0.0137	*0*.*0020*	0.0113	*0*.*0068*	0.0313	*0*.*0000*	−0.0002	0.7341	0.0013	0.3871	0.0012	0.3206
D6S1017	0.0037	0.1154	0.0001	0.6745	0.0038	0.0675	−0.0005	0.8710	0.0179	*0*.*0010*	0.0051	0.1095	0.0035	0.1623	−0.0010	0.9453	0.0008	0.5181	−0.0025	1.0000	0.0042	*0*.*0420*
D6S474	0.0026	0.2024	0.0061	0.0675	−0.0009	0.9961	0.0012	0.2903	0.0007	0.5386	0.0007	0.5161	−0.0009	0.9101	0.0032	0.1799	−0.0011	0.9326	−0.0018	1.0000	−0.0010	1.0000
D9S1122	0.0028	0.2072	−0.0029	1.0000	0.0066	*0*.*0156*	0.0002	0.6520	−0.0014	0.9902	0.0000	0.7038	0.0068	*0*.*0450*	0.0012	0.4350	−0.0005	0.8065	−0.0008	0.9071	0.0015	0.2317
D1S1627	0.0144	*0*.*0059*	0.0337	*0*.*0000*	0.0306	*0*.*0000*	0.0218	*0*.*0000*	0.0164	*0*.*0068*	0.0196	*0*.*0020*	0.0299	*0*.*0000*	0.0316	*0*.*0000*	0.0142	*0*.*0127*	0.0007	0.4712	0.0055	*0*.*0274*

### 3.5. Genetic distances and population differentiations

For further study, the pairwise *D*_A_ and *F*_st_ values between the Kyrgyz and the other reference groups were calculated, which are not only presented in table 4 but also shown with a clustered bar chart (electronic supplementary material, figure S2). The largest two values of *D*_A_ were observed between the Kyrgyz and the Yi group (0.0356) and then the Russian group (0.0252), whereas the smallest two were found between the Kyrgyz and the Uygur group (0.0083), and then the Kazak group (0.0097). Correspondingly, the *F*_st_ values were ranged from 0.0002 (between the Kyrgyz and the Kazak group) to 0.0350 (between the Kyrgyz and the Russian group), which were basically in line with the *D*_A_ values. The parameters in table 4 directly demonstrated that the studied Kyrgyz ethnic group and the two Central Asian populations (Kazak and Uygur ethnic groups) had close genetic relationships, which contrasted with the Yi and Russian groups, with relatively distant genetic relationships.

**Table 4.** The pairwise *D*_A_ and *F*_st_ values between the Kyrgyz group and other 11 reference groups were calculated based on 21 non-CODIS STR loci.

indexes	Yi	Russian	Salar	Tibetan	Tujia	Bai	Mogolian	Ningxia Han	Guanzhong Han	Kazak	Uygur
*D*_A_	0.0356	0.0252	0.0238	0.0211	0.0202	0.0194	0.0192	0.0178	0.0151	0.0097	0.0083
*F*_st_	0.0267	0.0350	0.0117	0.0127	0.0194	0.0105	0.0091	0.0112	0.0122	0.0002	0.0034

### 3.6. Phylogenetic analysis of 12 populations

**Figure 2.**
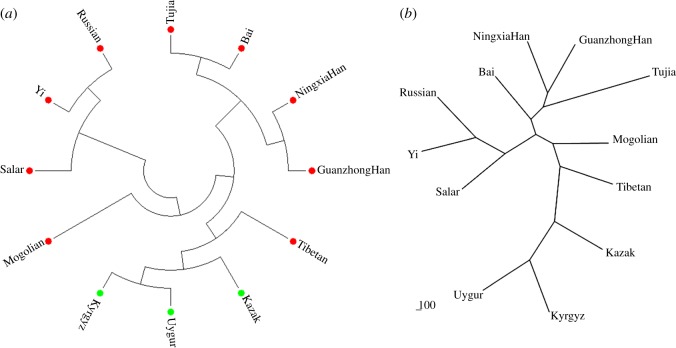
NJ trees for the Kyrgyz and the 11 reference populations were constructed by Mega v. 6.06 based on *D*_A_ values (*a*) and by Phylip v. 3.69 based on allelic frequencies (*b*).

The NJ tree was constructed by MEGA v. 6.06 based on *D*_A_ values represented in figure 2*a*. From this figure, the Kyrgyz group labelled by a green dot was first clustered with the Uygur and the Kazak group (green dots), and then with six East Asian ethnic groups marked by red dots: Tibetan, Tujia, Bai, Ningxia Han, Guanzhong Han and Mogolian group; however, there was another cluster that consisted of three East Asian ethnic groups marked by red dots: Yi, Russian and Salar groups. In figure 2*b*, another NJ tree was constructed by Phylip v. 3.69 based on gene frequencies, which is consistent with figure 2*a*. In the case of using different software based on different data formats, the three groups from Central Asia were clustered together in the two NJ trees, which revealed that they had stronger genetic relationships than the other populations from East Asia.

### 3.7. Multidimensional scaling based on the pairwise *F*_st_ values

**Figure 3.**
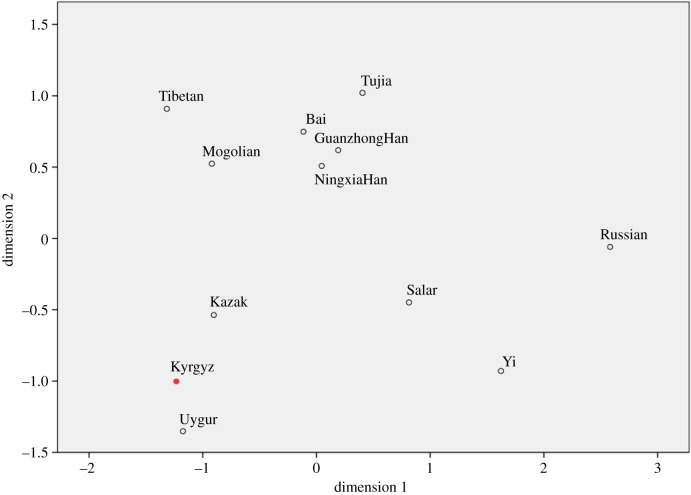
A two-dimensional MDS plot showing the genetic relationships of 12 different groups based on the pairwise *F*_st_ values.

As shown in figure 3, MDS was performed among the 12 populations based on the pairwise *F*_st_ values and the studied Kyrgyz ethnic group was marked with a red colour. The result indicated that the 12 populations could be divided into three parts: Tibetan, Mogolian, Bai, Tujia, Guanzhong Han and Ningxia Han ethnic groups were clustered in the upper quadrant; Salar, Russian and Yi ethnic groups in the lower right quadrant; whereas, Kazak, Uygur and the studied Kyrgyz ethnic groups in the lower left quadrant. Compared with the nine East Asian ethnic groups, the Kyrgyz ethnic group had even more intimate relationships with the Kazak and Uygur ethnic groups, which indicated that the Kyrgyz group probably had close genetic relationships with the two ethnic groups from Central Asia.

According to historical records, from the Western Han Dynasty to the middle of the Qing Dynasty, the Kyrgyz group, mainly stemming from the Yenisai River to the Tianshan Mountains and Central Asia, experienced five westward migrations which were basically facilitated by warfare [30]. In this study, the Kyrgyz group residing in the southwestern part of the Xinjiang Uygur Autonomous Region, China, broadly assimilated the culture of the western regions after long-term dwelling with the Uygurs, Kazaks and other ethnic groups in Xinjiang.

## 4. Conclusion

In short, the 21 non-CODIS STRs were detected in 307 individuals from the Kyrgyz ethnic group to evaluate the forensic effectiveness of these loci and to explore the genetic background of the Kyrgyz group. The present result indicated that these non-CODIS loci could be well applied in individual identification and kinship testing for their high level of genetic polymorphisms. The studies on population genetics also demonstrated that the Kyrgyz ethnic group had more similar consanguineous relationships with the Kazak and Uygur groups than the other reference groups to some extent.

## Supplementary Material

Supplementary Figure 1

## Supplementary Material

Supplementary Figure 2

## Data accessibility

Our data are deposited at Dryad: (http://dx.doi.org/10.5061/dryad.h4c25) [31].
