# Supplementary figures and images for: Forensic efficiency estimate and phylogenetic analysis for Chinese Kyrgyz ethnic group revealed by a panel of 21 short tandem repeats
Source: R Soc Open Sci. 2018 Jun 13;5(6):172089. doi: 10.1098/rsos.172089 (PMC6030347; doi:10.1098/rsos.172089)

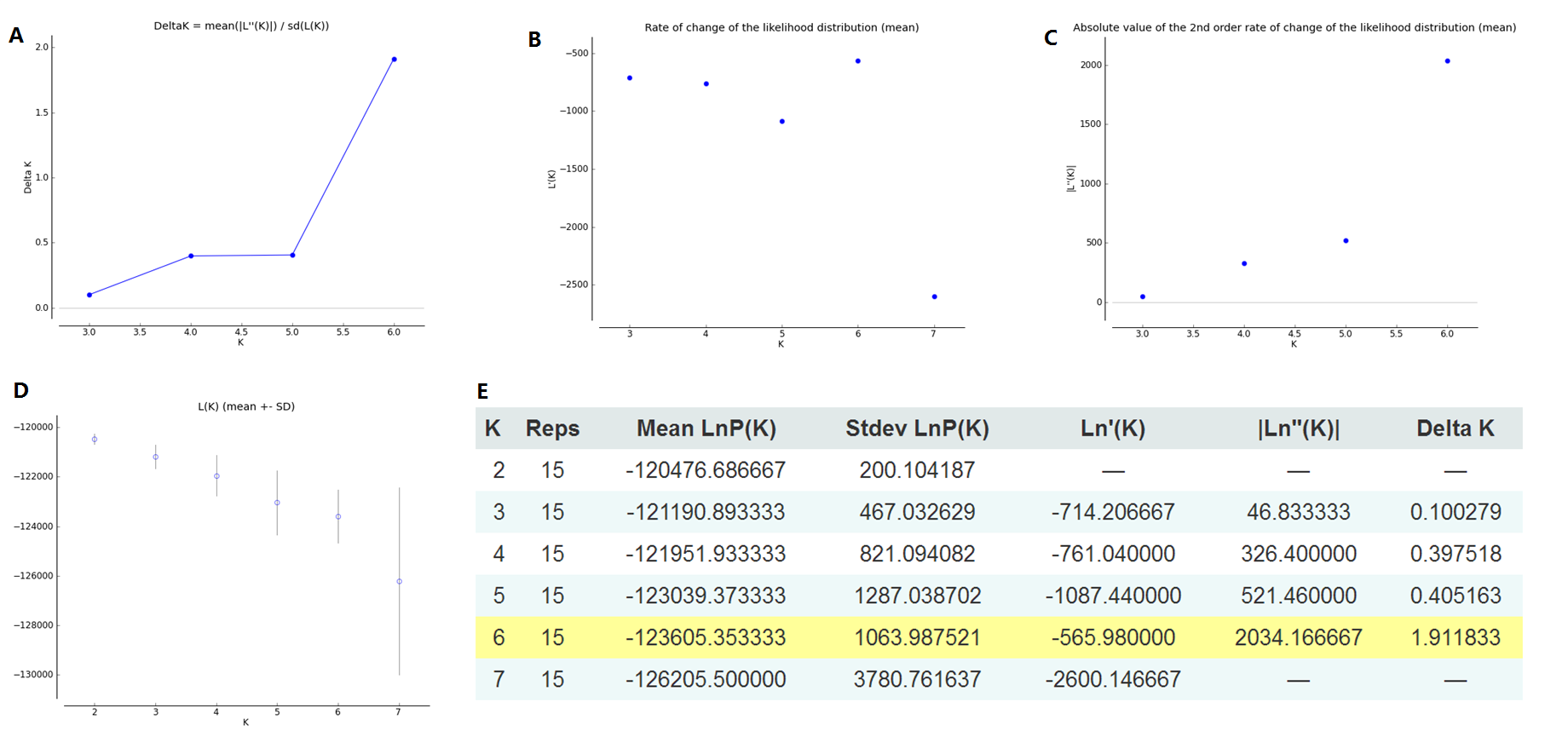

Supplement: Supplementary Figure 1 [file rsos172089supp1.png]

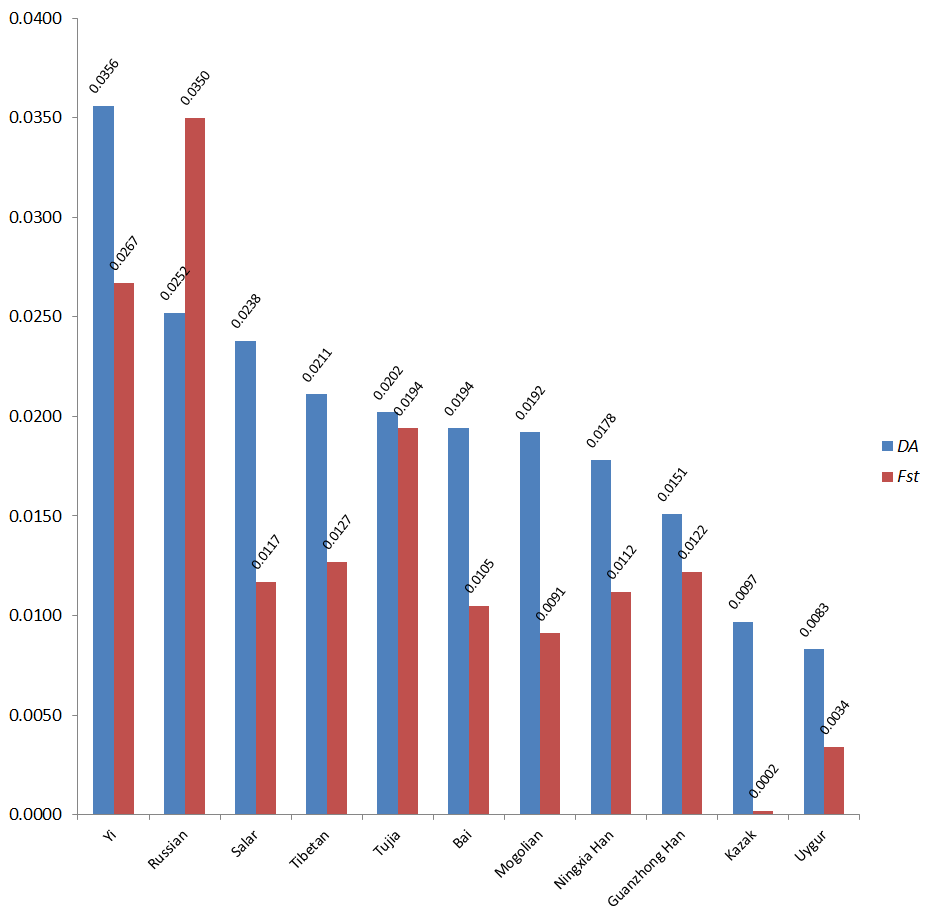

Supplement: Supplementary Figure 2 [file rsos172089supp2.png]
